# Effect of Glu12-His89 Interaction on Dynamic Structures in HIV-1 p17 Matrix Protein Elucidated by NMR

**DOI:** 10.1371/journal.pone.0167176

**Published:** 2016-12-01

**Authors:** Yuta Konagaya, Rina Miyakawa, Masumi Sato, Akimasa Matsugami, Satoru Watanabe, Fumiaki Hayashi, Takanori Kigawa, Chiaki Nishimura

**Affiliations:** 1 Faculty of Pharmaceutical Sciences, Teikyo Heisei University, Nakano, Tokyo, Japan; 2 Division of Structural and Synthetic Biology, RIKEN Center for Life Science Technologies, Yokohama, Kanagawa, Japan; 3 Laboratory for Biomolecular Structure and Dynamics, RIKEN Quantitative Biology Center, Yokohama, Kanagawa, Japan; Hokkaido Daigaku, JAPAN

## Abstract

To test the existence of the salt bridge and stability of the HIV-1 p17 matrix protein, an E12A (mutated at helix 1) was established to abolish possible electrostatic interactions. The chemical shift perturbation from the comparison between wild type and E12A suggested the existence of an electrostatic interaction in wild type between E12 and H89 (located in helix 4). Unexpectedly, the studies using urea denaturation indicated that the E12A substitution slightly stabilized the protein. The dynamic structure of E12A was examined under physiological conditions by both amide proton exchange and relaxation studies. The quick exchange method of amide protons revealed that the residues with faster exchange were located at the mutated region, around A12, compared to those of the wild-type protein. In addition, some residues at the region of helix 4, including H89, exhibited faster exchange in the mutant. In contrast, the average values of the kinetic rate constants for amide proton exchange for residues located in all loop regions were slightly lower in E12A than in wild type. Furthermore, the analyses of the order parameter revealed that less flexible structures existed at each loop region in E12A. Interestingly, the structures of the regions including the alpha1-2 loop and helix 5 of E12A exhibited more significant conformational exchanges with the NMR time-scale than those of wild type. Under lower pH conditions, for further destabilization, the helix 1 and alpha2-3 loop in E12A became more fluctuating than at physiological pH. Because the E12A mutant lacks the activities for trimer formation on the basis of the analytical ultra-centrifuge studies on the sedimentation distribution of p17 (Fledderman et al. Biochemistry 49, 9551–9562, 2010), it is possible that the changes in the dynamic structures induced by the absence of the E12-H89 interaction in the p17 matrix protein contributes to a loss of virus assembly.

## Introduction

The HIV-1 p17 matrix protein (p17) is a multifunctional protein[1], essential for viral infection. The p17 protein binds to different types of proteins and macromolecules including the cytokine receptor[2,3], membrane[4], RNA[5,6], and calmodulin[7]. To complete these functions, a flexible structure at the binding sites may be important to recognize other molecules[8]. Dynamic structures on a nano-pico second time scale can be analyzed using NMR techniques including T_1_ and T_2_ relaxation, and hetero-nuclear Overhauser effect (NOE) studies[9]. The motions can be monitored on a much slower time scale by amide proton exchange, which corresponds to the millisecond time scale[10].

The structure of p17 comprises five α-helices as determined by NMR[11] and was shown to be similar to that of interferon-gamma[12]. p17 is synthesized as part of the intact HIV-1 Gag polyprotein, which includes 6 proteins[13]. p17 is located at the N-terminal end of this precursor protein and is immediately followed by the HIV-1 p24 capsid protein (p24)[14,15]. Modification with a myristyl group at the N-terminus is essential for the membrane-binding activity of p17[16]. To perform biological functions, it is necessary for an HIV protease to cleave the precursor protein prior to its maturation[17].

The solution structure and dynamics of p17 connected to the N-terminal domain of p24 were analyzed in its premature form[18]. The two connected domains comprised an N-terminal 283-residue fragment of the HIV-1 Gag polyprotein. NMR relaxation studies on this connected protein revealed that the structure of the C-terminal region (K110-Y132) in p17 was flexible even in the presence of p24 at the C-terminus of p17. A trimer of p17 with myristyl groups was shown to be the functional membrane-associated form by X-ray crystallography[19].

Mutagenesis studies on the structure-function relationship of p17 have been widely performed[20,21]. On the basis of the studies on the site-directed mutagenesis, it was shown that the trimer formation of p17 was in good agreement with the formation of virus-like particle[21]. The exposure of a myristyl group, which can be correlated to membrane-binding activity, has been tested using mutants including V7R, L8A, L8I, and L21K[22]. On the basis of the solution structure of p17, the putative existence of a salt-bridge between E12 and H89 was determined[11], but it was not clearly demonstrated[12]. An E12A mutant that was designed to eliminate this salt bridge was tested for trimer formation and virus release efficiency[23]. E12A retained similar efficiency for virus release compared to wild-type (WT) protein, whereas the E12A mutation abolished trimer-forming activity, even in the presence of a myristyl group at the N-terminus[23].

In the present study, first of all, the interaction between E12 and H89 in the p17 structure was confirmed by the chemical shift perturbation studies using E12A and L85A. Next, the dynamic structure of E12A was analyzed by T_1_, T_2_, and heteronuclear NOE experiments. In addition, fluctuations on a slower time scale were monitored by phase-modulated clean chemical exchange (CLEANEX-PM)[24] for the quick exchange of amide protons using E12A as well as L85A. Importantly, the existence of a more flexible motion at the regions of each loop for WT, compared to that of E12A, was observed through order parameter analyses. Furthermore, more significant conformational exchanges were observed at the α1–2 loop and helix 5 in E12A compared to WT. It was concluded that the formation of the trimer of p17 was either promoted by the flexible motions existing at loop regions in the WT protein or interrupted by unfavorable conformational exchanges in E12A.

## Materials and Methods

### Protein preparation

Recombinant WT p17 protein and two mutants, E12A and L85A, were produced for NMR studies. DNA encoding the WT sequence was purchased from Hokkaido System Science Co., Ltd. The DNA fragments, which contained an N-terminal His-tag, a tobacco etch virus protease cleavage site, and linker sequences, were cloned into the pCR2.1 (Invitrogen) expression vector to make a fusion protein[25]. QuikChange^TM^ (Stratagene) was used to mutate the DNA for the subsequent expression of mutant proteins. The ^15^N-labeled and ^15^N,^13^C double-labeled p17 proteins with N-terminal His-tags were synthesized using a cell-free protein expression system at RIKEN with isotope-labeled amino acids[26] and purified using affinity chromatography[27]. After His-tag purification of the protein, the N-terminal tag was cleaved with tobacco etch virus protease and removed with HisTrap and HiTrap SP columns (GE Healthcare). The WT and mutant proteins were purified on a HiLoad 16/60 Superdex 75 column (GE Healthcare). Protein concentrations for WT and mutant protein samples were determined from A280 values. The molar extinction coefficients for WT and mutant proteins were calculated based on the amino acid sequences.

All NMR samples were dissolved in deuterated 20 mM Tris-HCl buffer (Cambridge Isotope Laboratories, Inc., MA) containing 100 mM NaCl, deuterated 1 mM DTT, and 10% D_2_O. A centriprep cartridge (Millipore) was used for the buffer exchange.

### Backbone assignments for mutants

All NMR spectra were recorded on a Bruker Avance 600 spectrometer equipped with a triple-resonance CryoProbe. Backbone resonance assignments for the two mutants of p17 (E12A and L85A) were independently made at 25°C using 3D CBCA(CO)NH[28], HNCACB[29], HNCO[30], HN(CA)CO[31], HNCA[30], and HN(CO)CA[30]. The concentration of the double-labeled proteins for backbone assignments was approximately 0.8 mM. The values of the chemical shifts of α- and carbonyl carbons in the random coil were calculated as reported by Schwarzinger et al.[32].

### Amide proton exchange monitored by CLEANEX-PM

All NMR spectra for both amide proton exchange and dynamics studies were collected at 25°C with an ^15^N-labeled protein at a concentration of 0.3 mM. The CLEANEX-PM heteronuclear single quantum coherence (HSQC) experiments were recorded with various mixing times (5, 10, 15, 20, 25, 50, and 100 ms) for the amide proton exchange. The spectra were collected without the mixing times as a reference. In order to calculate the rate constant for amide proton exchange (k_ex_)values, the signal intensities for the amide protons of each residue were curve fitted with an exponential function. The errors were calculated as standard errors of the fitted parameters. Finally, the protection factors were calculated by using the intrinsic exchange rates (k_int_) based on the primary sequence[33]. The average values of the rate constants for the fast exchangeable amide protons were calculated to compare the exchange rates between WT[34] and E12A at pH 7.

### Relaxation experiments and calculation of the order parameter

^15^N relaxation experiments, including T_1_, T_2_, and heteronuclear NOE, were conducted with pulse sequences as described by Farrow et al.[35]. The T_2_ data were acquired with relaxation delays at 17, 34, 51, 68, 85, 119, 170, and 220 ms. A 1–ms delay was employed between 180 pulses for the Carr-Purcell-Meiboom-Gill pulse train. The T_1_ data were recorded with relaxation delays at 10, 70, 150, 250, 370, 530, 760, and 1150 ms. Both T_1_ and T_2_ experiments were performed with repetition delays of 1.5 s. The T_1_ and T_2_ relaxation data were curve fitted with exponential functions to calculate the R_1_ and R_2_ values, respectively. The errors were calculated as the standard errors of the fitted parameters. Heteronuclear NOE experiments were performed using a 3-s saturation period and a 3-s recycle delay with both saturated and unsaturated pairs. The errors in heteronuclear NOE were estimated from the baseline noise in the spectra.

All NMR spectra were processed using NMRPipe[36] and NMRView[37] for optimal visualization and spectral analyses. The NMR data for WT[34], including chemical shifts, amide proton exchanges, and relaxation studies, were used for the comparison of dynamic structures between the WT and mutant proteins.

The program TENSOR2 was used to perform a Lipari-Szabo-type analysis of T_1_, T_2_, and heteronuclear NOE data[38]. Efficient analyses of macromolecular rotational diffusion from heteronuclear relaxation data were performed [39] for the structure of 2h3f.pdb[40]. In the model-free approach, assuming anisotropic tumbling[41], the internal mobility of residues was characterized by the order parameter S^2^ for the dynamics of WT and E12A at pH 7[9]. Generally, using the anisotropic diffusion tensor makes the tendency of internal mobility simple. Monte Carlo simulations were performed for the fully anisotropic tensor model. Co-axial NH (distance N-H: 1.02 Å) and axially symmetric chemical shift anisotropy (CSA) tensor were hypothesized. In the case of the dynamic structure of E12A at pH 5.5, isotropic tumbling was assumed for the calculation. The data was not able to be fitted by the anisotropic model. The tau-c value (1.123 x 10^−8^ s) was set by the software for the calculation. The values of R_ex_ and tau-e were calculated for each residue to check the reliability of the calculation for the order parameter.

### Circular dichroism

Circular dichroism spectra for the urea denaturation of p17 were measured on a Jasco J-810 spectropolarimeter at 25°C and pH 7. The ellipticity of the proteins in 20 mM Tris-HCl buffer containing 100 mM NaCl and 1 mM DTT was recorded at 222 nm with a protein concentration of 10 μM in the presence of different concentrations of urea[42]. Individual samples were prepared for measurements of the curve during urea denaturation. The thermodynamic parameters, including the ΔG(H_2_O) and *m* values, were calculated based on the denaturation curves. ΔG(H_2_O) is an estimate of the values of ΔG in the absence of urea. *m* value is a measure of the dependence of ΔG on the urea concentration.

## Results

### Thermodynamic studies of the E12A mutant

Circular dichroism was used for monitoring the urea denaturation of p17[43]. In the absence of the denaturant, the values of the ellipticity, monitored at 222 nm, for the folded WT and E12A proteins were almost the same (S1 Fig). The stability of WT and E12A proteins was compared as a function of the concentration of urea used for denaturation at pH 7 (Table 1). The midpoints for the transitions of the urea denaturation curves (C_m_) were 4.38 M for WT and 4.34 M for E12A. The ΔG (H_2_O) values were calculated based on the denaturation curves as—4.85 ± 0.24 kcal mol^-1^ for WT and—5.92 ± 0.24 kcal mol^-1^ for E12A. The *m* values for WT and E12A were 1.11 ± 0.06 kcal mol^-1^ M^-1^ and 1.37 ± 0.06 kcal mol^-1^ M^-1^, respectively. The amplitude of the value of ΔG (H_2_O) of E12A was much higher than that for WT. Therefore, it was shown that E12A is slightly more stable than WT. The unfolding of E12A was more cooperative than that of WT.

**Table 1:** Thermodynamic parameters for the urea denaturation of p17.

	ΔG(H_2_O)	*m*	C_m_
	(kcal mol^-1^)	(kcal mol^-1^M^-1^)	(M)
WT	–4.85 ± 0.24	1.11 ± 0.06	4.38
E12A	–5.92 ± 0.24	1.37 ± 0.06	4.34

### Effects of the E12A mutation on the carbon and proton chemical shifts

**Fig 1:**
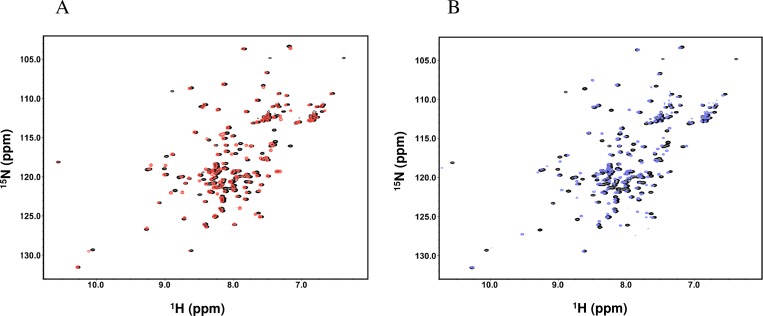
Superimposed HSQC spectra of p17 E12A (A: red) and L85A (B: blue) with WT (black) at pH 7 and 25°C.

**Fig 2:**
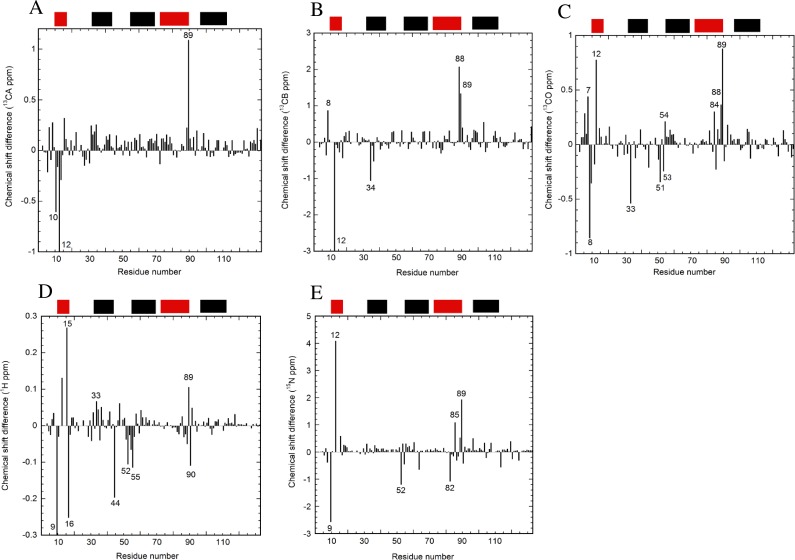
The differences in the chemical shifts between E12A and WT at pH 7. The differences in the chemical shifts of (A) CA, (B) CB, (C) CO, (D) H, and (E) N are indicated. Some residue numbers are shown. The values for residue 12 were out of the shown range and were -3.2 (A) and -9.9 (B), respectively. The value for residue 9 was -0.8 (D). The locations of the helices are shown at the top of each figure with black bars for helices 2, 3, and 5, and red bars for helices 1 and 4, based on the structure of p17 (PDB: 2H3F). The values of chemical shifts are shown for E12A (S1 Table).

The HSQC spectra are presented for WT and E12A at pH 7 (Fig 1A). The effects of the mutation were examined by comparing chemical shifts between E12A and WT. Changes in the chemical shifts were observed at the mutated region around A12 for CA, CB, CO, NH, and N (Fig 2). In addition, another change in the chemical shift was observed at V84-Q90. The chemical shift perturbations strongly suggest that an interaction between E12 and V84-Q90 (most likely H89) occurs. For CA, the values of residues for the entire region of the protein were nearly positive (Fig 2A), indicating a more helical structure in E12A compared to WT. Additional effects were observed around the region of T53 for CO, NH, and N (Fig 2C, 2D and 2E). Some differences in chemical shifts were also observed at H33 and I34 (Fig 2B, 2C and 2D). Based on the differences in the chemical shifts between the observed and random coil values, there were no global changes in the helical structures caused by either the E12A or L85A mutation (S2 Fig).

### Effects of the E12A mutation on dynamic structures

**Fig 3:**
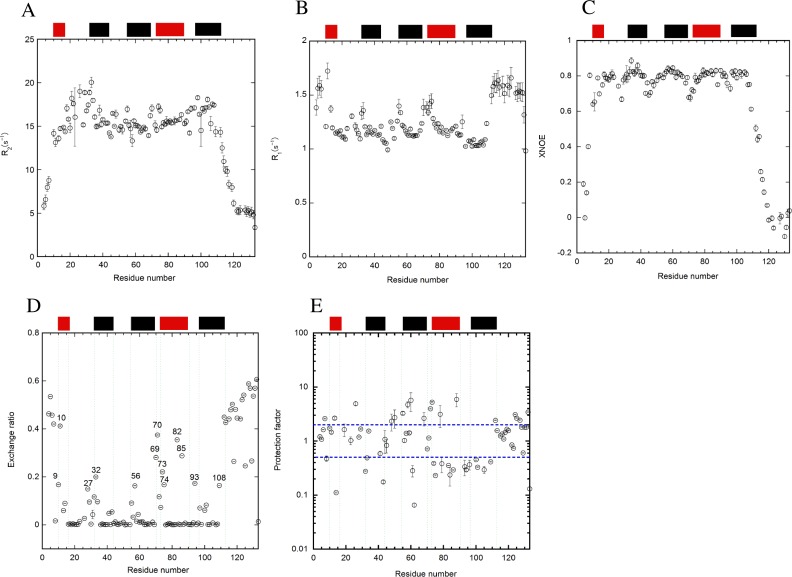
Relaxation studies on (A) T_2_ and (B) T_1_, and (C) heteronuclear NOE of E12A. The data was collected at pH 7 and 25°C. (D) The amide proton exchange was monitored by CLEANEX-PM at pH 7 and 25°C for E12A. The exchange ratios of amide protons for each residue were monitored by comparison with the intensities without the mixing time. The number of residues with a high exchange ratio is shown excluding the residues at the N- and C-terminal regions. (E) Protection factors were calculated based on the k_ex_ data of CLEANEX-PM in the same condition. Intrinsic exchange rates from the primary sequence were used for the calculation. The locations of the helices are indicated as bars at the upper part of the panel in a similar manner for the colors in Fig 2. The error bars are included in all figures.

The T_1_, T_2_, and heteronuclear NOE were collected for dynamic studies at pH 7 and the dynamics were analyzed by calculating R_1_, R_2_, and xNOE (Fig 3A, 3B and 3C). The average R_2_ value for the folded regions of E12A was approximately 15.5 (Fig 3A), and was similar to that of WT[34]. However, the values of residues located in the α12-loop and helix 5 in E12A were significantly higher than those in other regions. The values of some residues located in these regions were greater than 17.5. On the contrary, the value of residues in the mutated helix 1 was 14, which was lower than that of WT[34]. It was also shown in the R_1_ dataset that the pattern of the R_1_ data was opposite to that of the R_2_ data (Fig 3B), indicating the consistency of data between R_1_ and R_2_. In particular, the R_1_ values in helix 5 were slightly lower than those in the other helices. In xNOE data, the residues corresponding to each loop region revealed lower values than those of the helices, indicating more flexible structures in the loop regions than in the helical regions (Fig 3C). Interestingly, the average values of xNOE for the helical regions in E12A (0.81) were slightly higher than those of WT (0.79)[34].

### Calculations of order parameters (S^2^)

**Fig 4:**
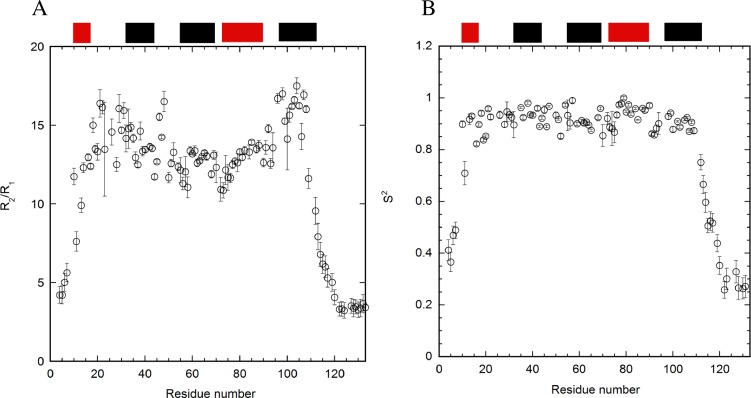
R_2_/R_1_ values (A) and order parameter S^2^ values (B) based on the relaxation studies of E12A at pH 7. The program TENSOR2 was used to analyze the T_2_, T_1_, and heteronuclear NOE data. The error bars and the locations of the helices are included. The tensor of the anisotropic diffusion to describe overall tumbling was used for the calculation at pH 7. The locations of helices are shown at the top.

The values of R_2_/R_1_ revealed the rotational correlation times for each residue of E12A at pH 7 (Fig 4A). These ratios are supposed to be independent of the internal mobility. Interestingly, the R_2_/R_1_ values were slightly higher in the α1–2 loop and helix 5 regions compared to other regions. This indicates the possible existence of chemical exchanges[44]. In contrast, the pattern of R_2_/R_1_ values for WT was somewhat complicated through the folded regions (S3A Fig).

Order parameters (S^2^) were calculated based on model-free analyses using the T_1_, T_2_, and heteronuclear NOE data (Fig 4B and S3B Fig). The tensor of the anisotropic diffusion to describe overall tumbling was used for the calculation at pH 7[45]. The values for Dx, Dy, and Dz were used as 0.115, 0.160, and 0.185 for E12A, and 0.112, 0.180, and 0.228 for WT, respectively. The analysis of the order parameters for WT demonstrated that the significant local minima of the values existed at each loop region (S3B Fig). In contrast, the difference in the order parameters for each residue was not so clear and monotonous between helices 1 and 5 for E12A (Fig 4B). The values of tau-e and R_ex_ were calculated to verify the order parameter for E12A (S4A and S4B Fig). This revealed that the pattern observed for R_2_/R_1_ (Fig 4A) was similar to that for R_ex_ (S4B Fig), reflecting the possibility for the chemical exchange.

Model 1 (S^2^), model 2 (S^2^, Tau-e), model 3 (S^2^, R_ex_), model 4 (S^2^, Tau-e, R_ex_), and model 5 (S^2^_s_, S^2^_f_)[45] were used for 16, 20, 19, 18, and 19 residues, respectively, to calculate order parameters for E12A at pH 7, and for 3, 7, 21, 26, and 13 residues, respectively, for the calculations for WT at pH 7.

### Effects of the E12A mutation on amide proton exchange

Quick amide proton exchanges were monitored by CLEANEX-PM (Fig 3D and 3E) for the millisecond time scale. As reported previously for WT[34], some areas including the N-terminal sections of helices 2 (K27-K32) and 4 (Q69-E74) revealed a higher exchange ratio for E12A (Fig 3D). In particular, the values of residues for the latter section were only slightly less than those at the N- and C-termini. Furthermore, the exchange ratio of the amide protons in residues in the region of the helix 1 around the mutated residue (A12) was significantly higher than that of other residues. In addition, some residues in helix 4, including I82 and L85, had the higher exchange ratios.

The average k_ex_ values for amide proton exchanges of the residues in the flexible region were calculated for WT and E12A at pH 7. Only reliable values of the exchange ratio that contained small errors with good fitting were used for the calculations. There were small differences in the rate constants between WT and E12A. The average values for WT was 0.040 ± 0.006 (ms^-1^) with 69 residues and 0.037 ± 0.007 (ms^-1^) with 75 residues for E12A. Calculations revealed that, except for the α4–5 loop near H89, the values of k_ex_ for WT were slightly higher than those for E12A. Sequence dependency was not included into the calculation of either exchange ratio or k_ex_. However, when comparing the values between WT and E12A, the sequences are identical with the exception of the mutated region.

By using the values of k_int_ based on the primary sequence[33], the protection factors for amide protons in each residue of E12A were calculated from k_ex_ (Fig 3E). In this figure, only the residues with fast exchangeable protons were observed for the short mixing time (5–100 ms). The protection factors at the regions for helices 4 and 5 around H89 seemed to be significantly lower compared to the other residues and those of WT (S3C Fig). The values of the protection factors were somewhat scattering throughout the entire molecule (Fig 3E).

### Effects of pH on the relaxation and amide proton exchange

**Fig 5:**
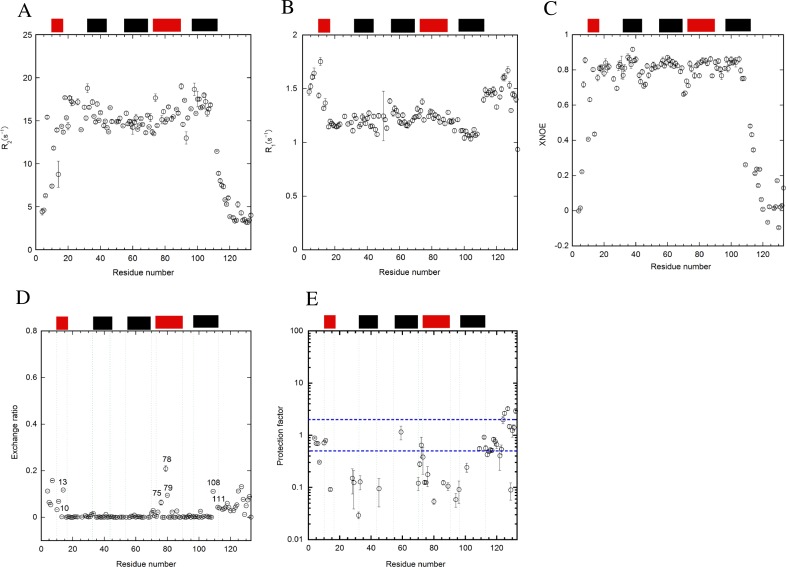
Relaxation data for E12A at pH 5.5 including (A) T_2_, (B) T_1_, and (C) heteronuclear NOE. (D) CLEANEX-PM data were collected at pH 5.5. (E) Protection factors were calculated based on the CLEANEX-PM data as a function of mixing duration. The error bars are included for each residue in all figures. The locations of the helices are indicated as bars at the upper part of the panel in the similar manner to Fig 2. The numbers of some residues, for which the values of the exchange ratio were high, are indicated in CLEANEX-PM data (D).

The relaxation studies for E12A were also conducted at pH 5.5 to achieve greater destabilization of E12A (Fig 5A, 5B and 5C). Comparison of relaxation data of E12A for pH 7 and 5.5 revealed small changes at the nano- and pico-second time-scale. On the basis of the T_2_ data, the R_2_ values for the residues in helix 1 and the α1–2 loop were slightly lower at pH 5.5 (Fig 5A) than those at pH 7 (Fig 3A). Furthermore, the values for the residues located at helix 1 were smaller for xNOE (Fig 5C) and larger for T_1_ (Fig 5B) at pH 5.5 compared to those at pH 7 (Fig 3B and 3C), suggesting that helix 1 fluctuated more at pH 5.5. In contrast, the R_1_ and xNOE values of the residues in all helices except helix 1 were similar between pH 5.5 and 7.

**Fig 6:**
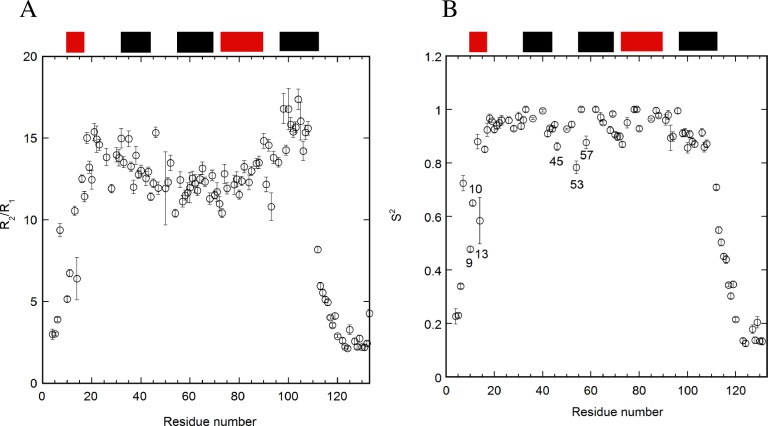
R_2_/R_1_ values (A) and order parameter S^2^ (B) based on relaxation studies of E12A at pH 5.5. The program TENSOR2 was used to analyze the T_1_, T_2_, and heteronuclear NOE data. For the dynamic structure at pH 5.5, isotropic tumbling was assumed for the calculation. The tau-c value was used as 1.123 x 10^−8^ sec for the calculation. The numbers of some residues, for which the values of order parameters were relatively low, are indicated in (B). The error bars and the locations of the helices are included.

The values of R_2_/R_1_ for E12A were slightly different between pH 7 (Fig 4A) and 5.5 (Fig 6A). Significant differences were observed at the N- and C-termini. The values at pH 7 and 5.5 were 4–6 and 2–4, respectively. The pattern of R_2_/R_1_ was similar between both pH conditions, and the values in the α1–2 loop and helix 5 were higher than those of other regions. In regards to S^2^ for E12A, the values for the residues in helix 1 (S9, G10, and L13) and α2–3 loop (A45, T53, and C57) were significantly lower at pH 5.5 (Fig 6B) than at pH 7 (Fig 4B). The values of tau-e and R_ex_ were calculated to verify the order parameter for E12A at pH 5.5 (S4C and S4D Fig).

At pH 5.5, models 1, 2, 3, 4, and 5 were used for 13, 9, 15, 8, and 35 residues, respectively, to calculate the order parameters for E12A. The number of residues for models 3 and 4 including R_ex_ were also low, as observed at pH 7 for E12A compared to WT.

In addition, the amide proton exchanges were monitored by CLEANEX-PM at pH 5.5 for E12A (Fig 5D). Even at pH 5.5, some residues indicated a higher exchange ratio at the N- and C-termini. Furthermore, the residues at helices 1 and 5 including G10, L13, Q108, and S111 revealed high ratios of exchanges. Interestingly, the amide protons of L75, L78, and Y79 showed higher ratios of exchanges compared to other residues.

The protection factors were calculated using the exchange data and k_int_. Fewer residues were available at pH 5.5 (Fig 5E) compared to at pH 7 (Fig 3E). The residues with lower protection factors were observed in the regions of helices 2 and 4 (Fig 5E).

### Effects of the L85A mutation on the chemical shift and amide proton exchange

**Fig 7:**
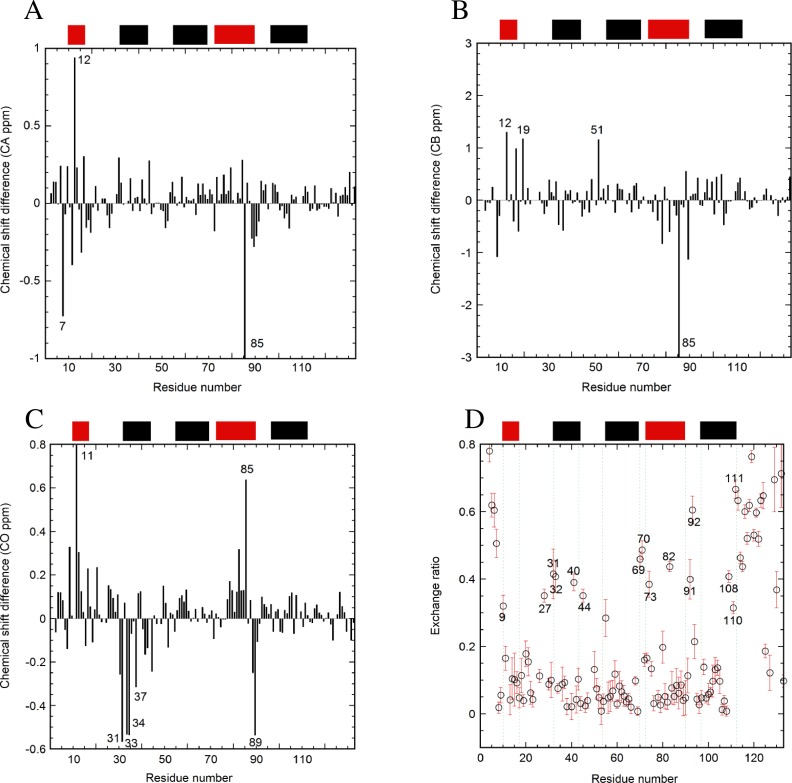
Effects of the L85A mutation on the chemical shifts of p17. The chemical shift differences of (A) CA, (B) CB, and (C) CO between L85A and WT at pH 7 are indicated. The residue numbers, which indicate large differences, are shown. The values of residue 85 were out of the shown range, and were -2.2 (A) and -25 (B). (D) CLEANEX-PM data for L85A at pH 7. The residue numbers, for residues that had quick exchange, are shown. The error bars are included. The locations of the helices are indicated as bars at the upper part of each panel. The values of chemical shifts are indicated for L85A and WT (S2 and S3 Tables).

In order to confirm the existence of an electrostatic interaction between E12 and H89, the L85A mutation was also produced in this study. The HSQC spectra were superimposed for L85A and WT at pH 7 (Fig 1B). Using the mutant, NMR studies were conducted to assess the chemical shifts of CA, CB, and CO (Fig 7A, 7B and 7C). Amide proton exchanges were monitored by CLEANEX-PM (Fig 7D). For the mutated region around A85, results indicated changes in the chemical shifts. In addition, the effects of the L85A mutation on the chemical shifts were observed for the residues of helix 1. Furthermore, the effects of the L85A mutation were observed around L31-A37 for CO (Fig 7C).

The effects of the L85A mutation on the protection of the amide proton exchange were plotted in Fig 7D. As with the WT, a high ratio of exchange was detected at K27-K32, Q69-E73, and R91-D93. In addition, residues including S9 and I82 had a higher ratio of exchange. Other significant changes in the pattern of amide proton exchanges were observed at E40 and F44. Finally, the amide protections of buried resides, which were not sufficient for a 100-ms exchange duration, were plotted around an exchange ratio of 0–0.1 for L85A at pH 7 (Fig 7D), whereas those of E12A (Fig 3D) and WT[34] at pH 7 were plotted at almost zero. Compared to L85A, the latter cases indicated a stronger protection for the exchange of amide protons throughout the molecule.

## Discussion

### Putative electrostatic interaction between E12 and H89

Based on the structure of p17, it was reported that a salt bridge is formed between H89 and E12[11] when H89 is protonated[23]. H89 is located at the end of helix 4, whereas E12 is located at the beginning of helix 1. However, it was not clear[12]. In the present study, the interaction between E12 and H89 was confirmed by the measurements of chemical shift perturbation (Fig 2). Since the pKa value of H89 was determined to be 6.5[23], a salt bridge between E12 and H89 is supposed to be formed in 50% of the population at pH 6.5. This interaction may stabilize the core structures composed of regions of helices 1 and 4.

At pH 7, the percentage of the WT protein population possessing a salt bridge between E12 and H89 was estimated to be 24% based on the ratio of protonated H89, which has a pKa of 6.5. However, it seemed that other electrostatic interactions, including a hydrogen bond, contributed to the interaction between these two residues without the protonation of H89. This was based on the chemical shift perturbation data as described below. In this study, the experimental plan was designed to use 1) WT at pH 7 with 24% of the proteins having an E12-H89 salt bridge (the most stabilized form), 2) E12A at pH 7 without the E12-H89 interaction, and 3) E12A at pH 5.5 (the most destabilized form). The E12A mutation did not cause a large structural change. Unexpectedly, the E12A substitution slightly stabilized the structure of p17 on the basis of the experiments of urea denaturation (Table 1).

### Changes in local structure caused by the E12A mutation

The changes in the chemical shifts observed at L8-A12, as well as V84-Q90, (Fig 2) were probably due to the loss of the electrostatic interaction like salt bridges, as well as hydrogen bonds between E12 and H89, caused by the E12A mutation. Thus, the existence of the electrostatic interaction in WT was indirectly confirmed by comparative studies on changes in the chemical shifts by the mutation at pH 7. The effects on the chemical shifts were observed quite locally, suggesting that no global structural change was induced even in the absence of the E12-H89 interaction.

Furthermore, it was shown that the structure fluctuated more in the helix 1 of E12A than in WT based on other dynamics experiments including the relaxation studies and amide proton exchange using T_2_ and heteronuclear NOE (Fig 3A and 3C) and CLEANEX-PM (Fig 3D). This was probably due to the absence of the electrostatic interaction between E12 and H89.

With the exception of the A12 and H89 regions, significant changes in the chemical shifts were only observed around T53 located at the N-terminus of helix 3. This was probably due to the formation of the hydrophobic core containing E12, H89, and T53, in the case of the WT protein. Without the interaction between E12 and H89 in E12A, the environment around T53 might be slightly changed.

Differences in the chemical shifts were also observed for the region of H33-I34 (Fig 2B and 2C). This was probably because the E12A mutation had a slight effect on the hydrophobic core. The hydrophobic interaction between helices 2 and 4 contributed to establishing the stable core structure including H33-T81 and W36-L78. Another possible mechanism is that the structural changes induced by E12A altered the environments of H33 including solvent accessibility.

### Effects of pH on the dynamic structures of E12A

At a lower pH, more of the protein population can have the salt bridge between E12 and H89 in the WT protein. However, only limited residues for fast exchange are available under the lower pH condition for CLEANEX-PM. On the other hand, the significant effects of the E12A mutation on the chemical shifts for the region around H89 were observed at pH 7, indicating the E12-H89 interaction (Fig 2). For these reasons, the dynamics data for WT and E12A were recorded at the physiological condition of pH 7.

Next, we intended to destabilize the structure of E12A in order to observe the more disordered structure. p17 may adopt a disordered structure based on its sequence[46]. We tested the further destabilization of E12A by changing the pH to 5.5 (Figs 5 and 6). However, even at pH 5.5, we were only able to observe more fluctuation in the regions of helix 1 and the α2–3 loop compared to pH 7, as determined by S^2^ values. Quick exchanges corresponding to the α3–4 loop region were observed in CLEANEX-PM for both WT[34] and E12A at pH 7 (Fig 3D). In contrast, quick exchanges were observed at L75-Y79 (N-terminus of helix 4) in E12A at pH 5.5 (Fig 5D). It is possible that the location of helix 4 in E12A is slightly shifted at pH 5.5, based on the CLEANEX-PM data comparing pH 7 (Fig 3D) to 5.5 (Fig 5D). A possibility is that the N-terminus of helix 4 is fragile at pH 5.5. The N-terminus of the helix is normally not stabilized by a hydrogen bond[47].

Other findings included the quick exchange at G10, L13, Q108, and S111 at pH 5.5 (Fig 5D). Residues at helix 1 including G10 and L13 as well as residues at helix 5 including Q108 and S111 indicated the significantly high exchange ratio even at pH 5.5 together with N-terminal and C-terminal tail residues compared to the other residues. The exchange ratios of these residues at pH 5.5 were relatively large compared to those at pH 7. Generally, the rate constant for the exchange at pH 7 should be more than 10 times larger than that at pH 5.5, because it is completely in proportion to the concentration of OH^-^ in solution. It is possible that the C-termini of helices 1 and 5 are less stable at the acidic pH.

The typical hydrogen deuterium exchange was employed for the p17 WT experiment[34]. At pH 7, it seemed that the number of probe residues were not sufficient for the experiment due to the quick exchange of protons. Therefore, the only probe residues available were located in the core region and buried. In this study using a CLEANEX-PM experiment, the amide protons, which are located in the loop regions, were focused on to compare the dynamic structures between E12A and WT. Furthermore, in the range of our experiments, the exchange ratio simply increased as a function of mixing duration, indicating that the reaction adopts the EX2 regime. The values of the protection factors seem to be slightly scattered perhaps due to the monitoring of the amide protons with fast exchanges

### The L85A mutation indirectly resulted in the breakage of the E12 and H89 interaction

The possible E12-H89 interaction could also be abolished by H89A. Originally, we intended to prepare the H89A mutant. However, since the H89A mutation led to a strong tendency for protein aggregation, the L85A mutant was designed and produced. The aggregation, with the H89G mutant, has been previously reported[23].

On the basis of both the chemical shift and CLEANEX-PM data, it was concluded that there could be no E12-H89 interaction in the L85A mutant (Fig 7) similar to that observed with the E12A mutant. The differences of the chemical shifts of CA, CB, and CO between L85A and WT (Fig 7A, 7B and 7C) were similar to those observed between E12A and WT (Fig 2A, 2B and 2C). In particular, the long-range effects of the L85A mutation in helix 4 were observed as differences in the chemical shifts at R7-E12 in helix 1 (Fig 7A, 7B and 7C). This was probably due to the indirect effects on the structure near H89 by the L85A mutation. The observed effects of the L85A mutation on the E12-H89 interaction were also supported by CLEANEX-PM data (Fig 7D). The quick exchanges of the amide protons at S9 (helix 1) and I82 (helix 4) were observed in CLEANEX-PM experiments for L85A.

The L85A mutation also caused other structural effects on regions including helix 2. These changes, monitored by the chemical shifts of CO (Fig 7C), were probably due to the effects of the structural changes near H33 by the L85A mutation. The interface structure between helices 2 and 4 can be tightly packed by the interaction of the side chains of H33 and T81. T81 is located close to the substituted A85.

### Stabilizing effect on the structure by E12A mutation

A less dynamic structure was unexpectedly detected through the entire molecule in E12A compared to in WT on the basis of the order parameter, heteronuclear NOE, and the average values of k_ex_ for quick amide proton exchanges. First, more flexible structures were observed for WT, as indicated by the values of the order parameters at each loop compared to other helical regions (S3B Fig). In contrast, the values of the order parameters for E12A were relatively unchanged from helix 1 to 5, including the loop regions, and had higher values (Fig 4B) than those of WT. Secondly, the average value of heteronuclear NOE for the structured regions of E12A was greater than 0.8 (Fig 3C), whereas that for WT was less than 0.8[34]. This suggested less fluctuation in structures throughout the molecule in E12A than in WT. Finally, the average rate constants derived from the amide proton exchanges of the entire flexible regions were lower in E12A compared to WT. Thus, the relaxation studies on the nano- and pico-second time scale, as well as the amide proton exchange with a milli-second time scale, revealed less fluctuation in the overall structure of E12A than WT. The mechanism of the stabilization caused by this breakage of the electrostatic interaction is unclear. However, it has been reported that the formation of a salt bridge is not necessary to stabilize protein structure[48,49]. One of the possible mechanisms for the destabilization induced by the formation of a salt bridge is the case of a buried salt bridge in the molecule[50,51]. It was reported that the existence of a hydrophilic charged residues in salt bridge buried in a molecule is unfavorable for thermodynamic stability. In this case, the hydrophobic interaction introduced by the mutations may more stabilize the molecule by the interaction between residues 12 and 89. A design for a stabilized p17 protein might be two combined mutations of E12L/H89F[52].

The results of relaxation and amide proton exchange are also consistent with the data from the thermodynamic studies on protein stability using urea denaturation of WT and E12A (Table 1). The amplitude of the value of ΔG (H_2_O) of E12A was higher than that for WT. Furthermore, differences in CA chemical shifts of E12A minus WT were mainly positive except in the mutated E12A region (Fig 2A), suggesting that a more helical structure was adopted in E12A compared to WT.

Thus, each region of the flexible loop in WT p17 may play an important role in the interaction with other molecules. The region including the α2–3 loop is related to virus infectivity[53]. Furthermore, the flexible regions including the α3–4 loop as well as the α1–2 loop are involved in the binding to RNA with phosphatidylinositol-(4,5)-bisphosphate[5]. In the presence of the membrane, p17 assembles as a hexamer of trimers with major structural changes[16,19]. The E12A mutation abolished the formation of a p17 trimer[23], which may be due to the formation of a less flexible loop in structure. In fact, both the α2–3 and α3–4 loops are located at the interface of the p17 monomer in the trimer[19].

### Conformational exchanges can contribute to negative effects on the interaction of p17 with other molecules

**Fig 8:**
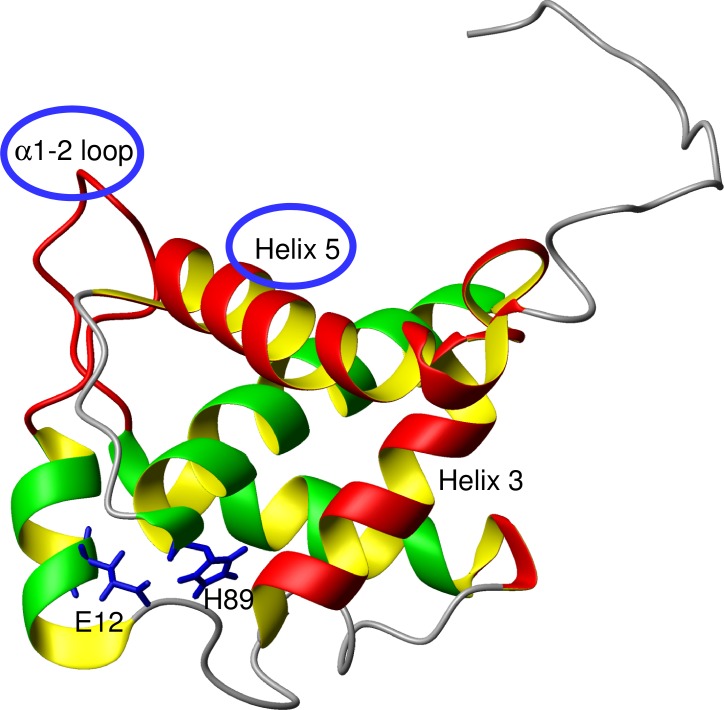
NMR structure of p17 (PDB: 2H3F). Red color: α1–2 loop, helices 3 and 5. The side chains for E12 and H89 are shown in blue. This figure was prepared using MOLMOL[62].

The R_2_/R_1_ data revealed that chemical exchange was mainly observed for the residues located in the regions of the α1–2 loop and helix 5 for E12A (Fig 4A). In contrast, the R_2_/R_1_ data for WT was complicated and showed no outstanding feature as a function of the residue number. The chemical exchanges were probably due to the occurrence of conformational exchanges at the α1–2 loop and helix 5 regions in E12A. This suggested that inhibiting the interaction between E12 and H89 contributed to the building of a few major exchangeable structures around the α1–2 loop and helix 5 in the E12A mutant. It may be concluded that the major conformational exchanges in either the highly basic region (α1–2 loop)[54] or helix 5 may not be suitable for trimer formation. The two regions are located in close proximity (Fig 8). In regards to the oligomeric structure of the matrix protein, this conformational exchange might contribute to the formation of a hexamer of trimers, which supports the interaction at the interfaces of the trimers.

### Biological relevance of the binding to the highly basic region and C-terminal helix and tail of p17

p17 is located at the N-terminus of the immature HIV-1 Gag protein[13]. p17 is targeted to the plasma membrane with the entire region of Gag polyprotein for its function[1]. The myristyl group, which is attached to the N-terminus of p17, adopts sequestered and exposed conformations[55] and plays a crucial role in binding to the plasma membrane of hematopoietic cells[40]. It was reported that p17 containing the myristyl group at the N-terminus, lacked a salt bridge between E12 and H89[56]. The observed dynamic structure containing conformational exchanges in this study might be due to the lack of a myristyl group in a construct of p17.

It was reported that a drastic structural change in helices 1 and 2 occurs upon binding to calmodulin[57,58]. It suggests the possibility that the dynamic structure of the α1–2 loop between helices 1 and 2 could be related to calmodulin binding. The region of L8-R43 in p17 was shown to be responsible for binding to calmodulin in other molecular biology experiments[59]. Furthermore, it was reported that the G11-K18 region in helix 1 was the p17 functional epitope[60]. The antibody, which recognized this region, intensely neutralized the detrimental function of p17 on the immune system. Furthermore, the highly basic region D14-L31 at the α1–2 loop is essential for interactions with acidic phospholipids in the membrane[22,54]. Thus, the highly basic region of p17 that we focused on is related to its function.

On the other hand, the conformational exchanges at helix 5 may control efficient interactions with other viral proteins. Studies on molecular dynamics of p17 revealed that helix 5 is partially unfolded[61]. Furthermore, it was shown that different reports on the length of the C-terminal helix 5 in p17 have been made due to the unstable structure[61]. In a previous study, we reported that the C-terminal region, including a partial helix 5 in p17, fluctuated more than the other helices[34]. This is functionally related to the contacts with other viral elements, including p24[18,43]. Therefore, the dynamic structure at the C-terminal region of p17 could contribute to viral maturation and assembly.

## Conclusion

More flexible structures were observed through the entire molecule in WT than in E12A. This flexibility may lead to the efficient formation of the p17 trimer. In contrast, a locally fluctuating structure including conformational exchange was observed only for E12A for the α1–2 loop and helix 5 regions. It was already shown that the binding sites for forming the p17 trimer are the α2–3 loop and α3–4 loop, which are separate from the regions of conformational exchange in the structure. The conformational exchange between the major structures might contribute to the negative effect on trimer formation. A salt bridge between E12 and H89 may function as an anchor to inhibit this unfavorable conformational exchange.

## Supporting Information

S1 Fig The curves for urea denaturation monitored by circular dichroism. Denaturation of WT (closed circle, blue line) and E12A (open square, red line) was monitored by molar ellipticity at 222 nm, pH 7.
(PDF)

S2 Fig Chemical shift differences between the values of observed (pH 7) and random coil structures. WT (A), E12A (B), and L85A (C) were calculated for differences based on the values of α–carbons.
(PDF)

S3 Fig The relaxation data monitored in WT[35] at pH 7 were analyzed by the program TENSOR2 assuming anisotropic tumbling. The values of (A) R_2_/R_1_ and (B) order parameter S^2^ were calculated. (C) Protection factors for WT were calculated on the basis of the amide proton exchanges derived from CLEANEX-PM data at pH 7. The locations of helices are indicated as black bars at the upper part of the panel. The error bars are included.
(PDF)

S4 Fig Model-free analyses were performed for E12A at pH 7.0 (A and B) and 5.5 (C and D). Tau-e (A and C) and R_ex_ (B and D) were calculated for both pH conditions.
(PDF)

S1 Table Chemical shift table for E12A at pH 7 and 25°C. (TXT)

S2 Table Chemical shift table for L85A at pH 7 and 25°C. (TXT)

S3 Table Chemical shift table for WT at pH 7 and 25°C. (TXT)
