# Supplementary figures and images for: Effect of Glu12-His89 Interaction on Dynamic Structures in HIV-1 p17 Matrix Protein Elucidated by NMR
Source: PLoS One. 2016 Dec 1;11(12):e0167176. doi: 10.1371/journal.pone.0167176 (PMC5132258; doi:10.1371/journal.pone.0167176)

## S1 Appendix

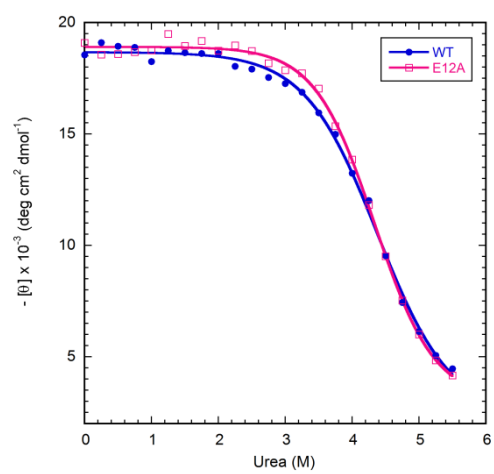

Supplement: S1 Fig — Denaturation of WT (closed circle, blue line) and E12A (open square, red line) was monitored by molar ellipticity at 222 nm, pH 7. (PDF) [file pone.0167176.s001.pdf]

S2

A

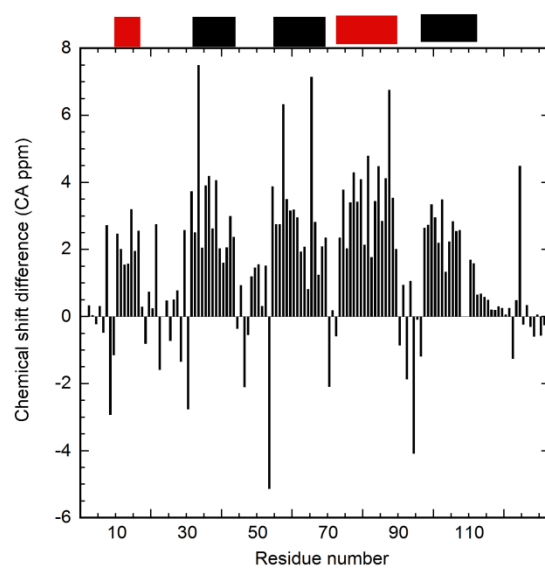

B

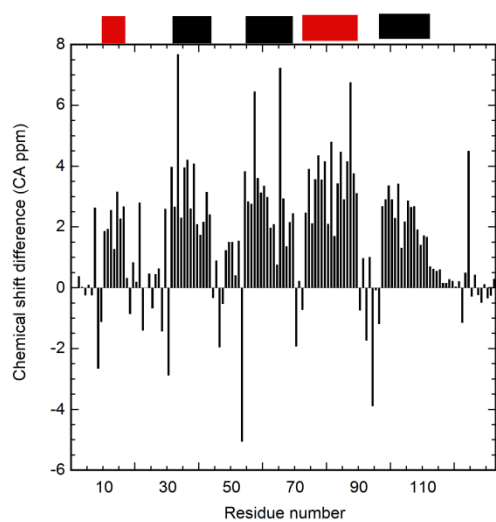

C

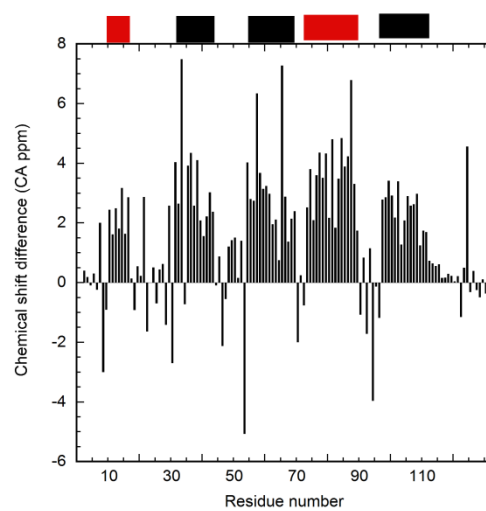

Supplement: S2 Fig — WT (A), E12A (B), and L85A (C) were calculated for differences based on the values of α–carbons. (PDF) [file pone.0167176.s002.pdf]

## S3 Appendix

(A)

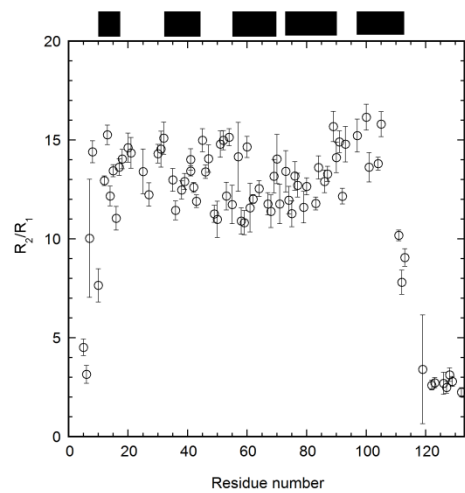

(B)

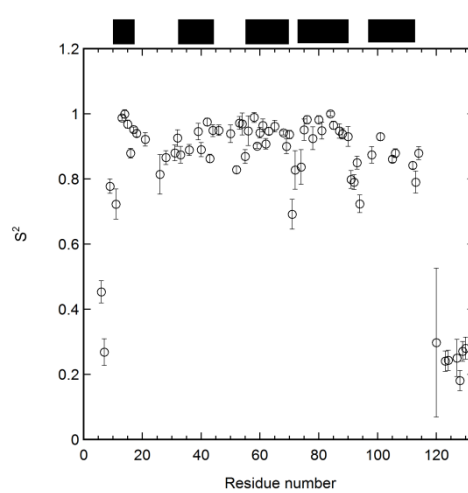

Supplement: S3 Fig — The values of (A) R2/R1 and (B) order parameter S2 were calculated. (C) Protection factors for WT were calculated on the basis of the amide proton exchanges derived from CLEANEX-PM data at pH 7. The locations of helices are indicated as black bars at the upper part of the panel. The error bars are included. (PDF) [file pone.0167176.s003.pdf]

## S4 Appendix

A

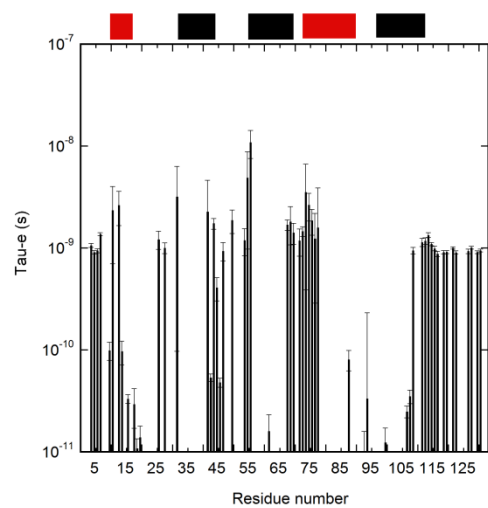

B

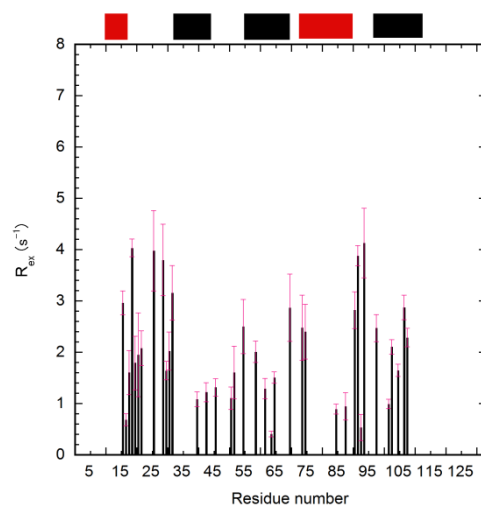

C

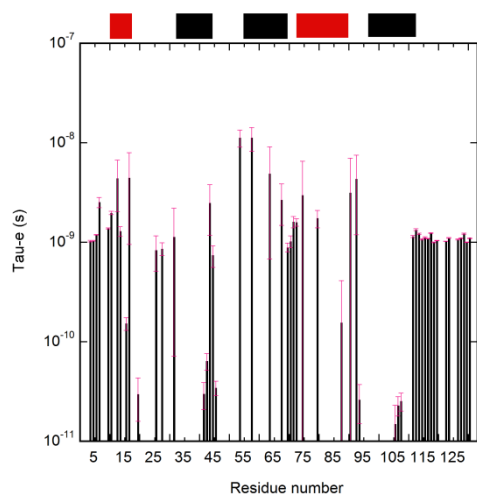

D

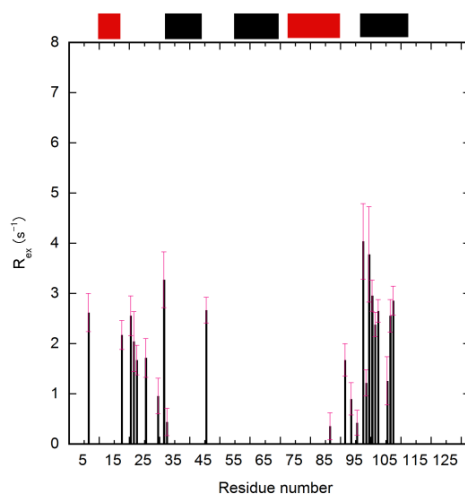

Supplement: S4 Fig — Model-free analyses were performed for E12A at pH 7.0 (A and B) and 5.5 (C and D). Tau-e (A and C) and Rex (B and D) were calculated for both pH conditions. (PDF) [file pone.0167176.s004.pdf]
